# Early Intervention in Unilateral Cerebral Palsy: Let’s Listen to the Families! What Are Their Desires and Perspectives? A Preliminary Family-Researcher Co-Design Study

**DOI:** 10.3390/children8090750

**Published:** 2021-08-30

**Authors:** Rocío Palomo-Carrión, Helena Romay-Barrero, Elena Pinero-Pinto, Rita-Pilar Romero-Galisteo, Purificación López-Muñoz, Inés Martínez-Galán

**Affiliations:** 1 Department of Nursery, Physiotherapy and Occupational Therapy, Faculty of Physiotherapy and Nursery, University of Castilla-La Mancha, 45071 Toledo, Spain; Rocio.Palomo@uclm.es (R.P.-C.); Purificacion.Lopez@uclm.es (P.L.-M.); Ines.Martinez@uclm.es (I.M.-G.); 2 GIFTO, Group of Research in Physiotherapy of Toledo, 45071 Toledo, Spain; 3 Pediatric Unit, Hemi-Child-Research (GIFTO), University of Castilla-La Mancha, 45071 Toledo, Spain; 4 Department of Physiotherapy, Faculty of Nursery, Physiotherapy and Podiatry, University of Seville, 41009 Seville, Spain; epinero@us.es; 5 Department of Physiotherapy, Faculty of Health Sciences, University of Málaga, 29016 Málaga, Spain; rpromero@uma.es

**Keywords:** co-design, early intervention, family involvement, infantile hemiplegia, therapist–family team

## Abstract

Cerebral palsy (CP) is a clinical diagnosis based on a combination of clinical and neurological signs, which occurs between the ages of 12 and 24 months. Cerebral palsy or a high risk of cerebral palsy can be accurately predicted before 5–6 months, which is the corrected age. This would allow the initiation of intervention at an early stage. Parents must be more involved in the development and implementation of the early therapy, increasing opportunities for parent–child interaction. The aim of this study was to learn from the perspectives of families with children under 12 months with unilateral cerebral palsy (UCP), what ingredients (barriers and facilitators) should be involved in early intervention so that we could co-design (researchers and families) a multidisciplinary guideline for a global intervention addressed to the needs of the child and the family. Semi-structured interviews were conducted at a time and venue convenient for the families. A total of ten families with experience in early intervention were invited to attend the interview with open questions: (1) What components should early intervention have for a baby diagnosed with UCP? (2) What components should early intervention have for the family? (3) What should the involvement of the family be in early intervention? (4) What barriers included in early intervention should be removed? From the data analysis, three key topics emerged and were subsequently named by focus group participants: (1) UCP early intervention components, (2) family involvement in early intervention of UCP, and (3) removing barriers and creating facilitators within early intervention. The participation of the families (mothers) in the co-design of the necessary ingredients within the scope of a multidisciplinary early intervention guide aimed at children with UCP under 12 months allows learning about their reality and not that of the therapist. The following list highlights the present barriers as perceived by the parents: intervention as spectators, therapeutic goals, clinic environment, and lack of empathy, and the possible facilitators determined by the parents during the implementation comprised teamwork, the family’s goals, motivation during the intervention, and learning at home. Thus, an early intervention program to improve global functionality should address family involvement through multidisciplinary coaching and the modification of the environment, encouraging family goals and family support through the family–therapist team.

## 1. Introduction

Cerebral palsy (CP) comprises a heterogeneous group of neurodevelopmental conditions that present mainly as movement and posture disorders accompanied by musculoskeletal problems, sensory impairments, perception, communication and behavior disorders, epilepsy, and cognitive and sensorial deficits [1]. Symptom onset occurs during early childhood, typically before 18 months of age [1]. The second most common CP subtype (after bilateral form), reported in around 25% of children with CP, is unilateral cerebral palsy (UCP), where one side of the body is affected as a consequence of brain damage that primarily affects one hemisphere [2]. “Developmental disregard” can appear in children with low performance, and it is defined as a failure to use the potential motor functions and capacities of the affected arm and hand for spontaneous use in daily life [3]. This phenomenon impairs the use of the affected hand and consequently disrupts bimanual co-ordination and interferes on the activities of daily living [4].

An early brain injury that might underlie cerebral palsy can lead to atypical brain development and reorganization, particularly during the first 2 years of life [5]. Cerebral palsy (CP) is a clinical diagnosis based on a combination of clinical and neurological signs, which occurs between ages 12 and 24 months [6,7]. Cerebral palsy or a high risk of cerebral palsy can be accurately predicted before 5–6 months corrected age. For early diagnosis, before 5 months corrected age, magnetic resonance imaging plus the General Movements Assessment or the Hammersmith Infant Neurological Examination are recommended; after 5 months corrected age, magnetic resonance imaging (where safe and feasible), the Hammersmith Infant Neurological Examination, and the Developmental Assessment of Young Children are recommended [7]. Early intervention is essential because infants who do not actively use their motor cortex risk losing cortical connections and specific function [8]. Furthermore, there is increasing evidence that the infant’s motor behavior, via discovery and interaction with the environment, controls and generates the growth and development of muscle, ligament, and bone as well as drives ongoing development of the neuromotor system [8,9]. UCP can be diagnosed at an early age [10,11], which would allow the initiation of the intervention at an early stage, reducing or preventing the limitations present in the affected side of the body that may affect their quality of life. The delay in diagnosis and intervention can cause extreme psychosocial stress for families [12]. When families are informed of a definitive diagnosis, they usually experience a wide spectrum of emotions [12]. They also experience stress from the lack of information available about the condition and the early intervention from the health services [13]. Chronic disease frequently affects the psychological functioning of parents and has an impact on the family [14], making it important to reduce the stress levels and to use family-centered practices [15].

Family-centered practices, which recognize the importance of including the family in the child’s care, have become the practice-of-choice in pediatric rehabilitation and early childhood intervention programs [15,16]. This involves the integration of parents of infants and young children with special needs as active participants in the intervention process, namely in goal setting, intervention planning, implementation, and evaluation [17].

For a successful parent-delivered intervention in young children with CP, the healthcare professional must acknowledge the importance of an environment-based support, mutual trust, and shared decision making [18]. Parents find it important to tailor the intervention to the child’s age, cognitive and physical disability, individual traits, and functional levels [18,19]. Parents also wish to modify the intervention to fit their differing parenting strategies and styles, personal beliefs, and environmental situations [20]. The factors influencing the success of parent-delivered therapy would be empowerment, motivation, and the reciprocal relationships between the parent, child, and healthcare professionals [21,22].

Thus, there are different methods of early intervention that are based on the active participation of the family to promote the motor functionality of children with cerebral palsy at an early age. In these methods, the importance of family training is critical, and they emphasize interventions based on family goals. Within this methodology we find Coping With and Caring for Infants With Special Needs (COPCA) for children with cerebral palsy being conducted by physiotherapists [23]. More specifically towards UCP, there is the Early Therapy in Perinatal Stroke (eTIPS) program—a parent-delivered, home-based therapy, which promotes the use of the affected side in an enriched environment and with family training. The eTIPS was developed within focus groups where a previous intervention design was discussed between families and health professionals [17]. That design was shaped and redesigned to mold it to the needs of the families who collaborated in its execution. Early intervention: sensorimotor development, attention and regulation, relationships, and therapist support (EI SMART) [24] was developed to encourage practitioners working with infants born preterm and other high-risk infants to work collaboratively with parents with the aim of optimizing and making early interventional support for infants and their families manageable as well as of identifying their own professional development requirements. It promotes multidisciplinary teamwork with the knowledge, skills, and attitudes to undertake appropriate assessments and work with parents to identify and address the needs of the child and family in a tailored programme [24].

Patient and Public Involvement (PPI) in the development of health research and early intervention is an essential element [25] The term PPI has been introduced to encourage the involvement of service users (families/children) and carers in health services [25,26]. Interpretations of families’ perceptions should not be made, but rather, they should jointly co-design the research and jointly interpret the results so that these are actually their experiences and not only those of the professionals [27]. Therefore, parents should be more involved and should work together with research teams on the development and implementation of early therapy, increasing opportunities for parent–child interaction.

Based on the importance of including the family from the beginning of the research to create the intervention design (co-design) and implementation of not only a motor approach, but a global approach for families and their children with UCP from an early age (before 12 months), we set ourselves the following objective:

To learn from families’ perspectives with children under 12 months with unilateral cerebral palsy what the ingredients (barriers and facilitators) that exist in early intervention are in order to co-design (researchers and families) a multidisciplinary guideline for a global intervention addressed to the needs of the child and the family.

## 2. Materials and Methods

### 2.1. Participants

Parents with children under 12 months of age who have been diagnosed with unilateral cerebral palsy were recruited by convenience from a pediatric hospital, where the health professionals have previously suggested the participants as possible good communicators of experiences or life stories due to their involvement in the process of the diagnosis and intervention of their children. This process was adopted to include families who had prior knowledge and experience in early intervention for children with UCP at an early age. It was deemed that those families would be ideally placed to comment upon factors and barriers which may be unique to study the necessary components in early intervention research and practice.

Inclusion criteria for the study were (1) Spanish families who had a child with unilateral cerebral palsy under 12 months with a minimum of 3 months having passed since the diagnosis and (2) who had interest in participating in early intervention development and motivation to build the components for its correct implementation. Individuals were excluded if they were unable to demonstrate teamwork competence or if they did not give the consent for the research and interview.

Written informed consent was obtained from all participants prior to interview.

The study complies with the guidelines determined by the Declaration of Helsinki as well as with the Spanish Law on Personal Data Protection and Guarantee of Digital Rights of December 2018. The study was also approved by the Ethics and Experimentation Committee of the University of Malaga (Ref No. 75-2020-H).

### 2.2. Study Design

Usually, the family receives the possible prognosis from the pediatric neurologist. When they are referred to early intervention, the health professional who accompanies the family asks them about their concerns and interests and also informs them about the child’s needs during the first year of life. The professional informs them about the possible development milestones that can be acquired and what possible interventions could be used, trying to use all of the possible assessments that facilitate what decisions should be made or what changes are necessary. However, on certain occasions, the professionals are the ones who decide the interventions to be applied without considering the needs that the family may have at that time and their wish to be involved in the implementation.

The main objective of early intervention is to optimize the development and well-being of children and their families, promoting their personal autonomy and participation within the natural environment [28]. Thus, early intervention provides a treatment within the cognitive area, motor skills, language, and communication to increase global development. When early intervention is performed in the hospital, the family has less involvement in the process, although the professionals can provide counselling and individual and/or group intervention for the families [29]. However, the time is limited due the number of children who are treated, and the families may feel that they do not receive sufficient and correct attention for the first stages of the early intervention. Therefore, we desired to determine these expectations and perspectives from families (using interviews) to co-design the specific ingredients that should be included at an early age according to their own experiences and knowledge.

Semi-structured interviews were conducted at a time and venue that was convenient for the families. This approach was chosen as it gives the families (the experience expert) the opportunity to lead topic discussions whilst offering the interviewer flexibility to probe interesting areas that arise and explain concepts in a variety of forms.

A total of ten families with experience in early intervention were invited to attend the interview with open questions (Table 1): (1) What components should early intervention have for a baby diagnosed with UCP? (2) What components should early intervention have for the family? (3) What should be the involvement of the family in early intervention? (4) What barriers included in early intervention should be removed?

All of the interviews were conducted by two health professionals: a physiotherapist and a psychologist specialized in pediatrics with more than 10 years of experience in early intervention and in unilateral cerebral palsy. The interviews were performed between November 2020 and January 2021 and lasted between 30- and 45-min. Analysis of the interview transcripts took place concurrently with data collection, and sample size was determined by the number of families recruited from a specific pediatric hospital that met the selection criteria.

### 2.3. Data Analysis

All of the interviews were audio recorded and were then transcribed verbatim. Transcripts were analyzed using a thematic approach guided by the proposed questions. In the first instance, each transcript was analyzed separately through a process of re-reading and descriptive coding followed by a more interrogative examination of the transcript at a higher conceptual level [30]. All emergent topics were placed in a list and were then reviewed to demonstrate if they could be included in the topics that had been established. To enhance scientific rigour, identified themes were presented and discussed during a focus group meeting assisted by two professionals. The focus group gave families the opportunity to build the content of each topic to be useful for families and professionals during the early intervention for unilateral cerebral palsy for children under 12 months of age. This process ultimately ensured that the findings reflected the participants’ perspective (mothers) and not the interpretations of the researchers [31].

## 3. Results

Parents (*n* = 10; age range: 30–40 years) met inclusion criteria, provided consent, and were interviewed for the study. All of them had previous experience with early intervention acquired in the hospital. All participants were mothers with higher education and city residents. In addition, all of them evaluated the interventions received from the professionals at the same pediatric hospital, whose guidelines used in the care of the child and family were the same for all participants and who participated in the family focus group discussion.

**Figure 1:**
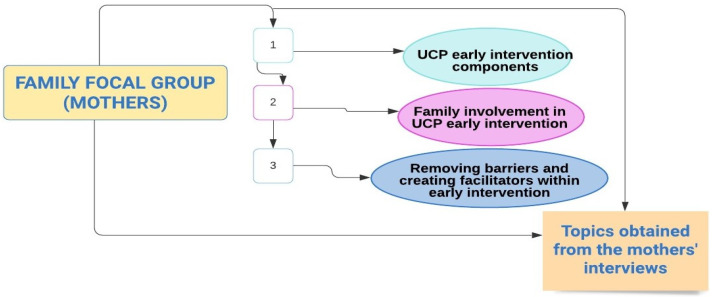
Topics obtained from mothers’ interviews.

From the data analysis, three key topics emerged and were subsequently named by the focus group participants (Figure 1):

### 3.1. UCP Early Intervention Components

**Figure 2:**
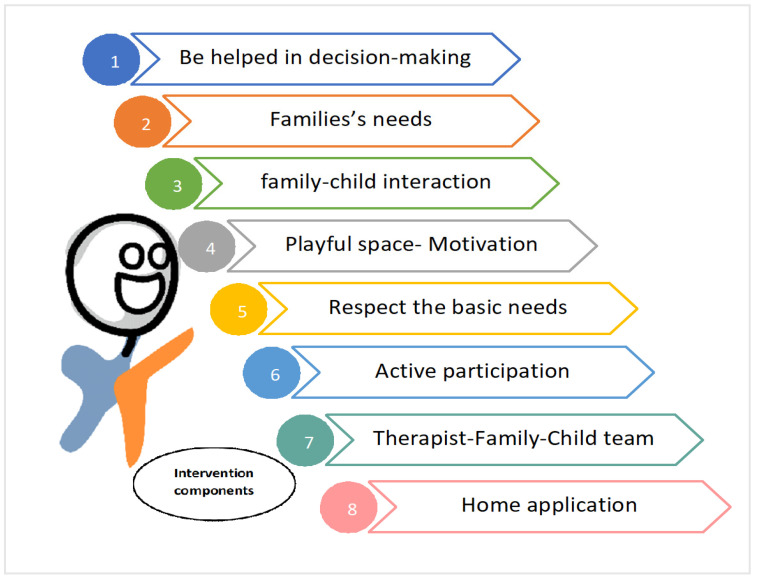
Components that should be included in UCP early intervention.

The mothers recognized the importance of participating in the process of choosing the intervention. The main requirement of the intervention had to consider the needs of the families, their fears, and insecurities. They indicated that an intervention based on family–child well-being should be offered and that their interaction should be promoted. The interventions had to offer a playful space for the child where motivation is present, meeting basic needs: sleep, bath, food..., etc. Intervention must allow the child to actively participate in the intervention without frustration, and the family should be able to be involved in that participation. The intervention must build a team: therapist–family–child. Intervention should be done at home so that the family can take advantage of the moments of the child’s daily routines to promote learning (Figure 2).

### 3.2. Family Involvement in UCP Early Intervention

The families stated that it was their right to be involved during the intervention at an early age. Families are the ones with whom children spend most of their time and know what their needs are at all times.

**Figure 3:**
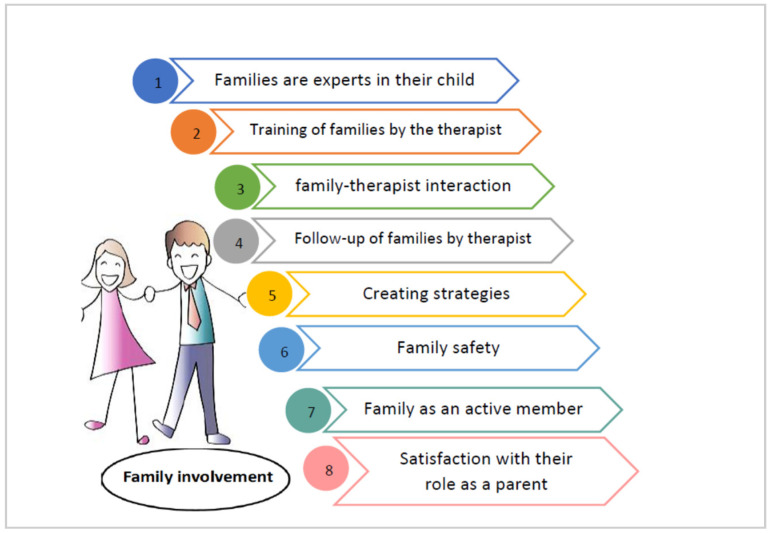
Components that should be included in family involvement in UCP early intervention.

This involvement must be acquired through training within the intervention program. It must be conducted by a therapist who provides the family with the adequate training in the management of their child. The involvement of the family would be possible with a follow-up of the therapist in which strategies can be created to introduce the intervention, resolve doubts, give support to the family, and modify the strategies that were not successful. In general, to allow the family to be an active member achieving self-confidence and satisfaction within their role as parents is what was desired (Figure 3).

### 3.3. Removing Barriers and Creating Facilitators within Early Intervention

**Figure 4:**
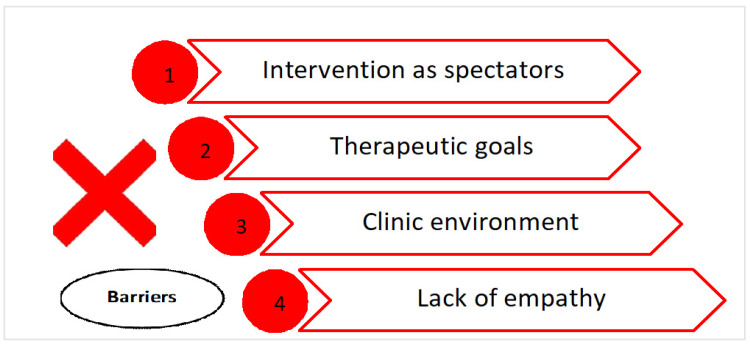
Barriers present in the UCP early intervention from the families’ perspectives.

**Figure 5:**
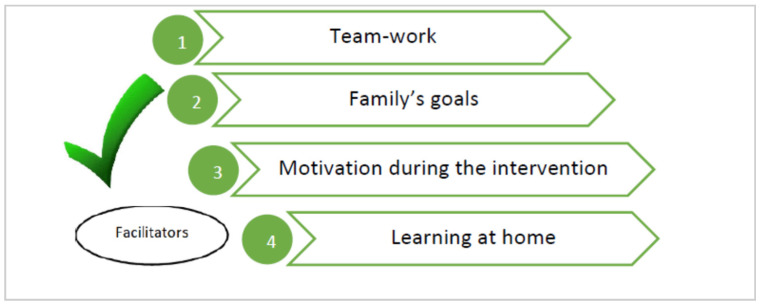
Facilitators present in the UCP early intervention from the families’ perspectives.

The families expressed their feelings of anguish and anxiety when they are treated as spectators of their children’s lives. All of them expressed their disagreement with therapists who do not ask them what their goals are, what they want to achieve, and what their concerns are. Another barrier is the clinical environment was the coldness of the sessions conducted in an unfamiliar place and the lack of empathy, on certain occasions, from the therapists themselves. The desires of the families were to create team groups, to set their own goals, to promote motivation within the intervention, to use play within the home, and to experience respect and empathy from the therapist towards the families, creating a team with a mutual interest in improving the quality of life of the child with UCP (Figure 4 and Figure 5).

## 4. Discussion

Using family involvement, the researchers are able to obtain the knowledge of the necessary ingredients that should be included in UCP early intervention and that would aid in the co-design (research and families) of a multidisciplinary guideline for a global intervention addressed to the needs of the child and the family. The active participation of families in the research allows researchers to get closer to their reality. Interviews as an exploratory method describe the key topics of family perceptions as part of PPI for early intervention in UCP. To our knowledge, this is the first time the perspectives and desires from families with children with UCP have been collected to co-design a multidisciplinary early intervention process using a research-based approach.

Families described the different components that should be present in the early intervention of children with UCP who younger than 12 months of age. It was curious that they highlighted the possibility of receiving help when making decisions about the therapeutic approach by health professionals (physiotherapists, occupational therapists, psychologists, and doctors) and actively participating in those decisions as well as decisions regarding the intervention place (home). Perhaps many families are subject to the professional’s decision, regardless of their needs. Unprepared clinicians also frequently ignore family’s emotional cues and miss opportunities to address their emotional needs [32]. Thus, a professional–family team should be built to use teamwork to prompt the use of recommended elements of a family-centered approach to improve the family’s perceptions of team communication with the clinical care team and increase the family’s perception of safety in care [33].

Exposure to greater challenges and stressors in family life as a consequence of having a child with a disability could negatively influence parenting practices. Parents of children with special care needs are still lacking the adequate supports necessary to help them at home in their role of intense parenting [34,35]. Meeting the needs of families for information as well as emotional and physical support and access to early intervention would reduce their stress levels [36,37] and would direct their perspectives to their own goals [38], which should be included within the perspective of families as facilitators.

Other facilitators were motivation in the therapy and learning at home. Integrating the concept of family-centered early intervention may enhance children’s motivation and families’ perceptions of their children’s capabilities [39,40,41]. It would allow a quiet place, where the times and basic needs of the child and the family are respected. The families would participate in their daily routines and would see the achievements according to their objectives, which gives rise to satisfaction and therefore improves the interaction in the game with the child and the handling of it. This interaction fosters motivation in the child’s participation and could reduce the frustration of intervening at imposed times and without the involvement of the family within a clinical setting [40,41]. Furthermore, the increased motivation may be beneficial for children to initiate movements at home and have more learning possibilities. A great health support for families of children with disabilities is to create opportunities for self-determination and participation by modifying the home environment [42]. These modifications provide children opportunities to facilitate control and engage in play, self-care, and household activities, creating accessible pathways in the house.

The involvement of the family was another of the key issues that were present through the need for training by the professional, their active involvement in the treatment, and creating a work team with follow-up to promote self-confidence. Family involvement as well as the barriers that may arise within early intervention would be conditioned by the characteristics of the families and their residence in urban or rural areas [43]. The availability and quality of specialized health services and providers are often inadequate to serve children in rural communities [44]. Differences in access to health care might be reflected in the type of attention the children receive. Accessibility factors, such as a lack of knowledge of health needs at early age and treatment options, inadequate funding, limited transportation, and social isolation also can create access barriers for families and children in rural areas [45]. Among parents of children with special health care needs, those living in rural areas could be more likely to report unmet health care needs caused by transportation and financial difficulties than those in urban areas [46]. In our study, only families from urban areas participated, so the ingredients they defined that should be included in early intervention might not be the same as those present in families from rural areas, where there are fewer resources. Therefore, the perspectives and desires between families in urban and rural areas could be different, and perhaps more barriers and challenges could be found for early intervention in rural areas. Thus, health professionals must strengthen quality, access, and collaboration within early intervention programs, parental support programs, medical care, and financial support programs to meet the needs of rural children and their families. Thus, families could be trained by a therapist to know and understand, through a dialogue with them, how to handle their child on certain occasions, how to cope with frustrating situations, or how to modify the environment to encourage learning and exploration and thus global development within their own routines [47]. Thus, family-centered treatment and coaching would be appropriate to promote adherence and satisfaction to the treatment that is aimed not only at the child’s motor development, but at their global development from an early age [47] and avoid families and their children not receiving the attention/sessions according to their needs, agreed upon by the family and the reference professional, due to the lack of resources because of living in a rural area. Health coaching is an educational, structured program defined as ‘a goal-oriented, client-centered partnership that is health-focused and occurs through a process of client-enlightenment and empowerment’ [48] to promote parental self-management and empowerment in the presence of the needs of their child with a chronic disability. Thus, families would obtain great knowledge into family-centered early coaching to encourage their own capacities to stimulate the infant’s global development during daily care in naturally occurring parenting situations [19,49].

The limitations of this study were the lack of the fathers’ involvement in the focus group, the lack of inclusion of different health professionals such as physiotherapist, occupational therapists, or psychologists as a focus group, and the small number and similar sociodemographic and cultural level of the families who participated in the co-design of the study. All of the participants belonged only to an urban environment. One of the possible consequences that this may imply is that rural families do not feel involved with the society in which they live, as reflected in some studies conducted in rural settings [50]. In contrast, another study concludes that many of the needs and preferences of parents living in rural areas are similar to those of other parents who do not live in these types of environments [51]. This dilemma might be resolved if parents are given the option of choosing where they want to receive the intervention for their children, in a hospital setting or natural setting, with a health professional support as reflected by Koçak et al. [52]. However, the first aim was to involve families to work on the research and to learn about their experiences and to involve families who were motivated to contribute to the development of early intervention and afterwards to include health professionals and families who reside in rural and urban areas to improve the acquired family-research co-design.

As strengths, it should be noted that it is the first co-design study that includes families from the beginning of the investigation to determine the components that should be present in the multidisciplinary early intervention of children with UCP under 12 months of age. Including their perceptions and desires as well as their real experiences places the therapist in their reality, allowing the parents to reach their goals and meet their expectations, not only the ones of the clinical professionals. Co-design allows the research to be disseminated from the participant’s own voice and not from the researchers’. Future lines of research will be the execution of the development and implementation of a multidisciplinary guide with the necessary ingredients of early intervention in children under 12 months of age to improve their global development and functionality through the inclusion of families and health therapists (physiotherapists, occupational therapist, and psychologists) in all phases of the research and to co-design multidisciplinary coaching for early intervention to ensure family involvement within the natural context.

## 5. Conclusions

The participation of families in the co-design of the necessary ingredients within a multidisciplinary early intervention guide aimed at children with UCP under 12 months of age allowed us to become aware of their reality and not that of the therapist. The parents highlighted the following barriers that are present in early intervention: intervention as spectators, therapeutic goals, clinic environment, and lack of empathy, and they identified the following possible facilitators within the implementation of early intervention: teamwork, family’s goals, motivation during the intervention, and learning at home. Thus, an early intervention program to improve global functionality should address family involvement through multidisciplinary coaching and the modification of the environment, encouraging family goals and family support, through the family–therapist team.

## Figures and Tables

**Table 1:** Questions and responses during the interview with ten families (mothers).

Open Questions in the Interview	Responses from Mothers during the Interview
What components should early intervention have for the baby diagnosed with UCP?	Mother 1. “Coaching families to understand how to handle the child at different times of the day”. Mother 2. “Increasing the functionality of our children. Respecting the periods of time in which the child is not attentive or is tired”. Mother 3. “Not generating frustration for the child or the family, that the child does not cry all the time and playing happens”. Mother 4. “That there is communication between the therapist and the family”. Mother 5. “For therapists to spend time with the family to listen to their problems because it is not easy to manage the situation of diagnosis and treatment”. Mother 6. “That families are able to make decisions together with therapists”. Mother 7. “Proposing targets that are not only those of the therapist”. Mother 8. “For the sessions not to be therapist–child, but for the family to be also a member”. Mother 9. “For families to understand what they want to do with our children”. Mother 10. “For their development to be promoted as best as possible and that there is not continuous crying during the intervention”.
What components should early intervention have for the family?	Mother 1. “Involving the family throughout the treatment process, not just telling them what to do”. Mother 2. “Reflecting together on what is best for our children, that we play an active role”. Mother 3. “We know our children, so we must be actively involved. We need to feel that what we do is okay and achieve satisfaction through common goals, not just the therapist”. Mother 4. “Allowing a pleasant situation to be created for the family and that frustration is not generated; our stress levels are very high; we do not know what will happen to our children”. Mother 5. An interaction to be established between the therapist (and) us as a family and to have an impact on our children with training in their management”. Mother 6. “For the intervention to be designed at home so that our time can be respected, with a follow-up by the therapist”. Mother 7. “For us to be able to accompany and decide on the treatment of our children and to not be judged”. Mother 8. “For us to be able to make use of our home as a means for the development of our children and to be taught some strategies to make correct use of them”. Mother 9. “For our needs to be understood, for the therapist to give us security in the handling of our children”. Mother 10. “For us to be able to design strategies or tools that are useful to improve the functionality of our children and to feel useful in the process”.
What should be the involvement of the family in early intervention?	Mother 1. “To be able to decide on the different tools or interventions that are going to be carried out with our children”. Mother 2. “We can guide the treatment from home to be able to use the moments when the child is calm”. Mother 3. “We know our children, and we need training to be able to offer them learning through our games”. Mother 4. “Deciding together with the professionals what to do, not just listening”. Mother 5. “To include ourselves in our children’s therapy and to learn to give them what they need from home, where they spend more time and with us”. Mother 6. “To have a say and not just listen to what the therapist tells us to do, establishing a joint dialogue for the betterment of our children”. Mother 7. “To be heard to plan the intervention from home and together”. Mother 8. “To use the knowledge we have of our children to promote their development and learning in a stable environment, such as home”. Mother 9. “It would be very good if a therapist–family team was created so that everyone’s well-being is enhanced and for it to be founded in a playful place”. Mother 10. “Our involvement must be active, not just listening because we know how to calm our children; we would need coaching to better interact with our children and to achieve the goals that we always set ourselves with motivation for all”.
What barriers included in early intervention should be removed?	Mother 1. “The clinical setting sometimes does not provide an opportunity for the family to participate”. Mother 2. “The clinical environment does not allow us to make use of our moments of greater tranquility and the hours and waits become very burdensome”. Mother 3. “The clinical environment and the therapists when they impose their own knowledge and do not give you the opportunity for dialogue”. Mother 4. “The therapist who does not listen to you and is in a clinical environment that is not motivating at all”. Mother 5. “Treatment at home may be better than in the hospital as it [hospital] does not offer many times when families can feel comfortable or calm”. Mother 6. “The lack of empathy of many professionals and their distance from families within the practice or hospital”. Mother 7. “Not being able to say what my son needs, the goals we want, and that this distance becomes greater at the practice”. Mother 8. “That the therapist is always the one who determines the moment in which the intervention should be carried out according to his goals in the hospital”. Mother 9. “The lack of empathy of the professionals who believe that they know everything at the hospital and are unaware of our problems, without giving us the opportunity to participate”. Mother 10. “Set goals to work at the hospital, but what happens at home? That stage ends, and we have to face a very difficult path without support”.

Mother 1: At the time of the interview, her baby was 4-months-old; Mother 2: At the time of the interview, her baby was 5-months-old; Mother 3: At the time of the interview, her baby was 5-months-old; Mother 4: At the time of the interview, her baby was 9-months-old; Mother 5: At the time of the interview, her baby was 8-months-old; Mother 6: At the time of the interview, her baby was 6-months-old; Mother 7: At the time of the interview, her baby was 7-months-old; Mother 8: At the time of the interview, her baby was 10-months-old; Mother 9: At the time of the interview, her baby was 6-months-old; Mother 10: At the time of the interview, her baby was 8-months-old.
